# Bone Density Variation in Rattails (*Macrouridae, Gadiformes*): Buoyancy, Depth, Body Size, and Feeding

**DOI:** 10.1093/iob/obac044

**Published:** 2022-10-16

**Authors:** Rene P Martin, Abigail S Dias, Adam P Summers, Mackenzie E Gerringer

**Affiliations:** Department of Ecology and Evolutionary Biology and Biodiversity Institute, 1345 Jayhawk Boulevard, University of Kansas, Lawrence, KS 66045, USA; Department of Biology, Sonoma State University, 1801 E. Cotati Ave., Rohnert Park, CA 94928, USA; Department of Biology and Friday Harbor Laboratories, University of Washington, 620 University Road, Friday Harbor, WA 98250, USA; Department of Biology and Friday Harbor Laboratories, University of Washington, 620 University Road, Friday Harbor, WA 98250, USA; Department of Biology, State University of New York at Geneseo, 1 College Circle, Geneseo, NY 14454, USA

## Abstract

Extreme abiotic factors in deep-sea environments, such as near-freezing temperatures, low light, and high hydrostatic pressure, drive the evolution of adaptations that allow organisms to survive under these conditions. Pelagic and benthopelagic fishes that have invaded the deep sea face physiological challenges from increased compression of gasses at depth, which limits the use of gas cavities as a buoyancy aid. One adaptation observed in deep-sea fishes to increase buoyancy is a decrease of high-density tissues. In this study, we analyze mineralization of high-density skeletal tissue in rattails (family Macrouridae), a group of widespread benthopelagic fishes that occur from surface waters to greater than 7000 m depth. We test the hypothesis that rattail species decrease bone density with increasing habitat depth as an adaptation to maintaining buoyancy while living under high hydrostatic pressures. We performed micro-computed tomography (micro-CT) scans on 15 species and 20 specimens of rattails and included two standards of known hydroxyapatite concentration (phantoms) to approximate voxel brightness to bone density. Bone density was compared across four bones (eleventh vertebra, lower jaw, pelvic girdle, and first dorsal-fin pterygiophore). On average, the lower jaw was significantly denser than the other bones. We found no correlation between bone density and depth or between bone density and phylogenetic relationships. Instead, we observed that bone density increases with increasing specimen length within and between species. This study adds to the growing body of work that suggests bone density can increase with growth in fishes, and that bone density does not vary in a straightforward way with depth.

## Introduction

Deep oceans make up the largest environment on Earth and are home to a diversity of animals that have adapted to abiotic factors dramatically different from surface conditions. As ocean depth increases, light levels decrease, and by 1000 m most light from the surface is filtered out (e.g., Marshall 1960; Randall and Farrell 1997). Water temperature drops within the first 1000 m to ∼4°C and remains steady around 0–3°C in the bathyal, abyssal, and hadal zones (e.g., Randall and Farrell 1997; Priede 2017). Hydrostatic pressure increases by 0.1 MPa every 10 m, reaching 110 MPa in the deepest parts of the ocean and >80 MPa at the greatest depths bony fishes are believed to inhabit (e.g., Yancey et al. 2014; Priede 2017; Gerringer et al. 2021a). Succeeding in these extreme deep-sea conditions has required fishes to evolve a range of adaptations across almost every organ system (e.g., Gibbs and Somero 1989; Somero 1992; Gillett et al. 1997; Randall and Farrell 1997; Morita 2008; Partridge et al. 2014; Yancey et al. 2014; Priede 2017; Gerringer et al. 2017a; Marranzino and Webb 2018; Martin et al. 2022). To survive in these deep, low-light conditions, fishes have evolved large and/or dorsally directed eyes (e.g., Barreleyes (Opisthoproctidae); Warrant and Locket 2004; Partridge et al. 2014) and the ability to see wavelengths of red bioluminescent light (Dragonfishes (Stomiidae); Randall and Farrell 1997). Some fishes, like the grideye fish (Ipnopidae), have lost complex eyes altogether, relying on other strategies to find food in the dark (Warrant et al. 2003). Other deep-sea fishes use enhanced mechanoreception to sense their surroundings. For example, some dragonfishes (Stomiiformes) have increased superficial mechanosensing neuromasts compared to their shallower-living relatives (Marranzino and Webb 2018). Frigid deep-sea temperatures can affect the response times of fish, and swordfish (*Xiphias gladius* Linnaeus 1758 (Xiphiidae)) evolved the ability to heat their eyes and brain to maintain temperature while making daily excursions into the deep sea to hunt (Carey 1982). Additionally, under high hydrostatic pressure maintaining buoyancy can become challenging, especially for fishes that use swim bladders due to the high compressibility of gasses (Somero 1992; Priede 2018) posing another obstacle for fishes invading the deep sea.

Swim bladders are gas-filled organs used for buoyancy, hearing, and sound production. They allow fish to maintain neutral buoyancy at a specific depth, reducing the amount of energy needed to swim by allowing a horizontal thrust vector rather than one that counteracts the downward force of negative buoyancy. Swim bladders are lightweight and supported by internal gas pressure that usually matches the pressure of the surrounding water (e.g., Priede 2018). Fishes can change swim-bladder volume as needed with changes in depth and pressure. Weight and density of gasses in the swim bladder increase under pressure, so fishes living in deep-sea habitats under high hydrostatic pressure may have a reduced ability to use swim bladders as a buoyancy aid (e.g., Randall and Farrell 1997; Priede 2018). Not all fishes rely on swim bladders to maintain buoyancy. Many have evolved an array of alternative methods to aid in buoyancy and successfully move through the water column and deep sea. Fast-moving fish species that travel large distances and are constant swimmers have countered swim bladder pressure issues by either reducing their swim bladders or losing them completely (e.g., Scombridae (tunas and mackerel) Denton and Marshall 1958; Dagorn et al. 2000; Rooker et al. 2008). Other fishes have replaced the gas in their swim bladders with lipids (e.g., Myctophidae (*Stenobrachius leucopsarus* (Eigenmann and Eigenmann 1890), *Diaphus theta* Eigenmann and Eigenmann 1890); Marshall 1960; Priede 2017). Because lipids and fats are less dense than water, sequestering lipids in the body can provide additional buoyancy in the water column (e.g., Eastman and DeVries 1982; Phleger 1998; Eastman and Sidell 2002). Decreasing high-density body structures can also increase buoyancy either by decreasing the total high-density tissue amounts or by decreasing tissue density (e.g., Drazen 2007; Gerringer et al. 2017a). Some of the highest density tissues in fishes include proteins in lateral muscles and mineralized skeletal tissue (bone; Denton and Marshall 1958). Bone is a heterogeneous material made of both mineral (calcium hydroxyapatite, CaHA, Ca_5_(PO_4_)_3_(OH)), and organic (e.g., collagen, proteins, water) components. The hydroxyapatite content of vertebrate skeletal tissue varies by species and by individual bone. Some snailfishes have <10% mineralization in their lower jaws (Gerringer et al. 2021b); whereas, the stapes of the human ear is ∼98% mineralized by dry weight (Boskey 2013). There is evidence for decreasing bone density, or demineralization, in the icefishes (notothenioids; Eastman and DeVries 1982), bristlemouths (*Gonostoma*; Denton and Marshall 1958), and snailfishes (Liparidae; Gerringer et al. 2021b). There are thousands of species of deep-sea fishes, but there are a limited number of studies focused on buoyancy adaptations. One previous study investigating depth-related buoyancy adaptations in snailfishes (Gerringer et al. 2021b) found that deeper living species had lower-density skeletons. We hypothesized that there may be other unidentified groups of deep-sea fishes that possess decreased skeletal mineralization as an adaptation to supplement buoyancy under increasing habitat depth and high hydrostatic pressures.

Fishes may have lower bone density at greater depths due to biological factors other than maintaining buoyancy. These other biological factors may obscure assessment of the trends in skeletal demineralization. Bones provide multiple functions including attachment points for muscles, defense, and overall support. The strength and density of the skeletal structures may be constrained by their association with functions like feeding and swimming. Fishes feed (e.g., biting, ram, suction) and swim (e.g., thunniform, labriform, tetraodontiform) in a variety of ways, and variation in the mechanical loading of different skeletal elements may alter their resulting density. Fishes of various sizes may also vary in their skeletal densities, with a general trend of increasing skeletal density with growth within a species (Hamilton et al. 1981). These biological constraints on bone density should be considered when assessing any trends associated with abiotic factors and adaptations such as those tied to high hydrostatic pressure and buoyancy.

Here, we investigate trends of bone mineralization with increasing habitat depth and other factors, including body size, by assessing a group of fishes called the rattails (family Macrouridae), marine fishes that are known to occur from near the ocean surface down to abyssal depths (Cohen et al. 1990). Rattails, (macrourids) are an abundant benthopelagic clade of fishes composed of 369 described species (Fricke et al. 2020). Rattails have a global biomass estimated at 1.5 × 10^7^ tonnes (Gage and Tyler 1991), a worldwide distribution, and with species’ depth occurrences ranging from near the upper continental shelf (e.g., *Coelorinchus aconcagua* Iwamoto 1978, maximum depth 450 m; Cohen et al. 1990) to depths greater than 7000 m (*Coryphaenoides yaquinae* Iwamoto & Stein 1974; Linley et al. 2016). Most species occur between 200–500 m (Priede and Froese 2013). *Coelorinchus* (122 described species) and *Coryphaenoides* (66 described species) are two of the largest genera in the Macrouridae (Priede 2017; Froese and Pauly 2019). Species in the genus *Coryphaenoides* are known to be some of the deepest-living rattails, boasting an average minimum depth of 1,103 m (Priede 2017) and a maximum known depth of 7,012 m (*Coryphaenoides yaquinae*; Linley et al. 2016). Most rattails glide continuously near or just above the sea floor, foraging most notably on crustaceans and other fishes, but many species are known to be scavengers (e.g., Drazen et al. 2008; Drazen and Sutton 2017; Priede 2017). The sheer abundance of rattails and their benthopelagic behavior makes macrourids an important connection between the pelagic and benthic systems in the deep sea worldwide (e.g., Drazen et al. 2008).

Rattails display multiple adaptations to maintain buoyancy in the deep ocean. *Coryphaenoides yaquinae* possesses gelatinous tissues that are buoyant in seawater (Gerringer et al. 2017a). Crabtree (1995), Drazen (2007), and Schwarzhans (2014) found evidence for watery muscles in multiple species of rattails, suggesting reduced density in their muscle tissue. While assessing rattail osteology, Gregory (1933) and Schwarzhans (2014) noted a “parchment-like texture” in rattail skulls and Günther (1887) found that mucosal chamber bones expand into thin lamellae and lose their firmness. These observations suggest possible demineralized skeletons in rattails, a pattern that may be prevalent throughout the group. By assessing bone density variation across species occurring at differing habitat depths, we can explore whether demineralization is occurring as an adaptation to remaining buoyant in the water column under higher hydrostatic pressures.

One way to measure bone density is through micro-computed tomography (micro-CT) scanning, a common technique used in the medical field and with increasing popularity in biological studies (e.g., Burrow et al. 2005; Puria and Steele 2010; Tapanila et al. 2013; Friscia et al. 2016; Di Santo 2019; Kruppert et al. 2020; Gerringer et al. 2021b; Morimoto et al. 2022). Density standards of known calcium hydroxyapatite values (phantoms) are included in scans and allow for the conversion of brightness values in voxels (3D pixels) in micro-CT images to the density of these standards. In this study, we use similar micro-CT scanning methods to investigate the variation in bone density of four different bones across a subset of rattails, assessing the possibility of bone demineralization as an adaptation to increasing buoyancy in this group.

Different bones may display different trends with increasing habitat depth depending on their function. In this study, we assess variation in density across four bones with varying biological functions, including the lower jaw (dentary, articular, and associated teeth), the pelvic girdle, the eleventh vertebrae, and the first dorsal-fin pterygiophore. First, the lower jaw might be constrained to a higher density due to its use in with feeding. Because prey structure and mechanical loading are not expected to vary with increasing habitat depth, we hypothesized that there would be little to no reduction in density in the lower jaw associated with depth. Second, the pelvic girdle, which supports the pelvic-fin musculature, may be less constrained by functional requirements as the pelvic fins of rattails are not used extensively in locomotion or as sensory systems. Thus, if demineralization is occurring in association with depth, it may present in bones with little-to-no mechanical loading, and we hypothesize a reduction in density of the pelvic girdle. Third, the eleventh vertebra is associated with mid-body muscle attachments. We hypothesize that intermediate mechanical loading on the vertebrae will reflect an average specimen bone density and thus decreases in the density of the vertebrae correlated with depth may reflect adaptations to increasing buoyancy. The fourth bone selected is the first pterygiophore which is associated with the dorsal fin. Due to its intramuscular position, we predict the first pterygiophore will reflect a decrease in bone density with depth if rattails are decreasing mineralization to increase buoyancy as it may be less constrained by functional use. We also investigate possible confounding results due to phylogenetic relationships (Felsenstein 1985) and assess whether there are changes in density due to specimen size. In this study, we address the following questions: (1) Does average bone density decrease with increasing habitat depth in rattails? (2) Does density vary across different bones in rattails? (3) Do different species of rattails exhibit different bone densities? (4) Is there a phylogenetic influence on bone density variation within rattails? (5) Is there variation in bone density by body length within and between rattail species? Observing trends in bone density variation within rattails occurring at differing habitat depths may allow us to test the hypothesis that deep-sea fishes are using demineralization as an adaptation to increasing buoyancy under higher hydrostatic pressures.

## Methods

### Specimen acquisition and micro-CT protocol

**Fig. 1:**
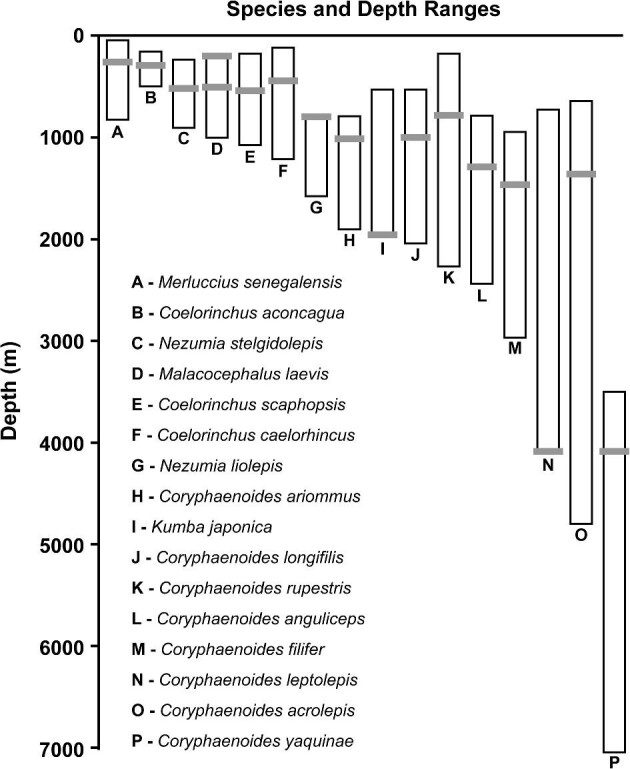
Depth ranges of macrourid fishes and a closely related merluccid used in this study. Ranges are described from the following studies: Cohen et al. (1990), Iwamoto and Sazonov (1994), Iwamoto and Schneider (1995), Hoff et al. (2015), and Linley et al. (2016), Cruz-Acevedo and Aguirre-Villaseñor (2020).

The 20 rattails used in this study (Table 1) were preserved museum specimens, fixed in 4% buffered formalin, and transferred to alcohol (75% ethanol or 50% isopropanol) for long-term storage. Specimens were loaned from the Scripps Institution of Oceanography (SIO; stored in isopropanol), the Burke Museum (UW; stored in ethanol), and the California Academy of Sciences (CAS; stored in ethanol). The 15 species in this study include representatives from the major macrourid genera *Coryphaenoides* and *Coelorinchus*, with additional representatives in *Nezumia* and *Malacocephalus.* These species also occur across a broad range of habitat depths, and the full depth range of the family (Fig. 1). To assess variation in bone density by specimen length within a species, we included two representatives of four different species (Table 1)*.* We also included one additional specimen from a closely related outgroup species of hake (*Merluccius senegalensis* Cadenat 1950, Merluccidae), a demersal fish that occurs in shallower waters. Institutional abbreviations and acronyms for museums and collections associated with all specimens and molecular samples follow Sabaj (2020).

**Table 1:** Specimens used in this study to assess bone density variation along with their museum ID, pre-anal fin length, depth of capture, maximum known habitat depth, date of collection, and Genbank accession numbers from previously published protein-coding sequences. For a list of studies associated with gene sequences, please refer to the methods text.

Genus	Species	Number of specimens	Museum ID	Pre-anal fin length (mm)	Depth of capture (m)	Max depth (m)	Date collected	COI	16S	RAG1
**Ingroup**	** **	** **	** **	** **	** **	** **	** **	** **	** **	** **
*Coelorinchus*	*aconcagua*	1	SIO 65-675	40	274	450	1965			
*Coelorinchus*	*caelorhincus*	1	SIO 65-367-62A	44	366	1250	1957	KF929773	FJ215119	FJ215213
*Coelorinchus*	*scaphopsis*	1	SIO 68-90	48	567	1158	1993	HQ127672	JX121806	
*Coryphaenoides*	*acrolepis*	1	SIO 71-141	91	1390	4700	1975	KF420483	EU099505	KX656582
*Coryphaenoides*	anguliceps*	2	SIO 59-265	55 ; 60	1366	2400	1976	MF956577		
*Coryphaenoides*	*ariommus*	1	SIO 72-183	95	1097	1860	1972			
*Coryphaenoides*	*filifer*	1	UW 41684	130	1406	2904	1996	KX656386	AY947842	KX656597
*Coryphaenoides*	*leptolepis*	1	SIO 91-152	85	4100	4100	1991	EU148126	KX656463	KX656606
*Coryphaenoides*	*longifilis*	1	UW 119661	65	1010	2025	2000	JF952709	AB512070	KX656608
*Coryphaenoides*	*rupestris*	1	SIO 74-183	91	800	1200	1974	KU943142	FJ215158	FJ215260
*Coryphaenoides*	yaquinae*	2	SIO 04-101 ; SIO 91-145	93 ; 110	4100	7012	2004 ; 1991	GU440292	KX656501	KX656642
*Malacocephalus*	laevis*	2	SIO 79-344 ; SIO 00-200	94 ; 109	229 ; 500	1000	1968 ; 1990	KU943142	FJ215158	FJ215260
*Nezumia*	*japonica*	1	SIO 77-157	28	2000	2000	1976			
*Nezumia*	*liolepis*	1	SIO 71-1	58	722	1655	1975	MF956872	KJ010551	FJ215283
*Nezumia*	stelgidolepis*	2	UW 153473 ; UW 153474	100 ; 110	534	909	2011	MF956879	KJ010688	
* *	* *									
**Outgroup**	* *									
*Lampichthys*	*procerus*							MF966948	AB042172	
*Merluccius*	*senegalensis*	1	CAS 235494	75	212	800	2012	GQ988403	DQ274040	
*Polymixia*	*japonica*	* *		* *				KF930291	DQ532939	JX189778

Here, we used micro-CT scanning following a similar protocol to Buser et al. (2020) to reconstruct high-resolution 3D images of macrourid skeletons. Micro-CT scans can separate hard tissues from soft tissues and are advantageous in studies using rare specimens, as a non-invasive procedure to obtain data on internal skeletal structure. Specimens were scanned at the Karel F. Liem Imaging Facility at Friday Harbor Laboratories, University of Washington using a Bruker Skyscan 1173 scanner with the following settings: 65 kV, 123 uA, 1 mm Al filter, 2k detector, with 0.3 rotation and voxel sizes ranging from 26.0 and 35.3 μm. Each scan included either two 7.5 mm or two 10.5 mm diameter Bruker phantoms made of calcium hydroxyapatite used for bone density calibration. Phantoms were of known densities: one 25% calcium hydroxyapatite and one 75% calcium hydroxyapatite. Note, the outgroup hake (*Merluccius senegalensis*) was not scanned with a phantom which is considered when discussing the results from this specimen. Reconstruction of micro-CT data was performed in NRecon v. 1.7.1.0 (Bruker 2017). Beam hardening was set to 30% and dynamic image range was set between 0.00 and 0.020 for each scan. The X/Y alignment, post alignment, and ring artifact reduction were chosen to optimize quality for each scan. If specimens were large or there were multiple specimens in a scan, scans were cropped in DataViewer v. 1.5.2.4 (Bruker) to reduce file size and allow for easier data manipulation during downstream analyses.

### Amira protocol and measuring bone density

**Fig. 2:**
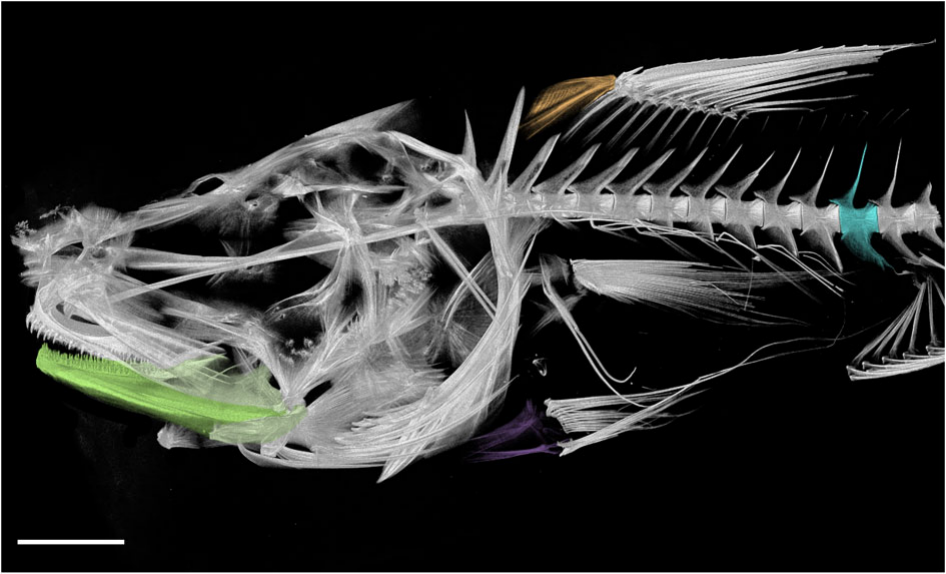
Scan of *Coryphaenoides leptolepis* (SIO 91-152) with the four assessed bones highlighted: green) lower jaw; orange) first pterygiophore; purple) pelvic girdle; turquoise) 11th vertebra. Scale bar represents 10 mm.

To assess bone density across rattails we used the software Amira (Stalling et al. 2005; Thermo-Fisher Software, MA, USA, 2022) to partition bones and acquire voxel brightness variation, later converted to relative bone density. Four different bones with different biological functions were chosen for this study including the lower jaw (dentary, articular, and associated teeth), the pelvic girdle, the eleventh vertebrae, and the first pterygiophore (Fig. 2). Bones were segmented in Amira and a low-end masking was used to obscure background material (e.g., cheesecloth, skin). Low-end masking values varied by scan, as some specimens and bones were of a lower density than others. Voxels of both phantoms and the four assessed bones were segmented and assessed individually in Amira for use in average brightness (density) analyses.

Brightness values measured from the voxels of each bone were related to density via a comparison to the average voxel brightness in the calcium hydroxyapatite (CaHA) phantom, a known-density standard. Using Amira, the average brightness values were calculated from the voxels for each partitioned bone, with brighter regions associated with higher density values. The mean pixel brightness (MPB) of the phantoms included in each scan were graphed against their CaHA percentage (a 25% phantom and a 75% phantom) to create a standard slope of the % CaHA for a given voxel brightness per scan. The resulting slopes of individual phantoms from each scan were used to calculate the % CaHA of individual bones. Although using two phantoms only allows for a linear relationship, Di Santo (2019) used similar methods with five phantoms and found a linear relationship between voxel brightness and hydroxyapatite content. The hake scan lacked associated phantoms but was scanned at the same settings for use in this study.

Micro-CT settings were optimized for scan quality resulting in slightly different settings among scans. Because each scan contained phantoms of known density, Amira colormap values were standardized across scans by converting the normal 0–255 BPM grayscale range to represent 10–90% calcium hydroxyapatite brightness using the following equations based on MPB and the VolrenWhite colormap (Equations 1 and 2).

(1) \begin{equation*} {Minimum\,Value{:}\left( {\frac{{\left( {MP{B}_{75} - MP{B}_{25}} \right)}}{{50}}} \right)* 10 + \left( {\frac{{MP{B}_{25}}}{{\frac{{(MP{B}_{75} - MP{B}_{25})}}{{50}}}}} \right)} \end{equation*}

(2) \begin{equation*} {Maximum\,Value{:}\left( {\frac{{\left( {MP{B}_{75} - MP{B}_{25}} \right)}}{{50}}} \right) *90 + \left( {\frac{{MP{B}_{25}}}{{\frac{{(MP{B}_{75} - MP{B}_{25})}}{{50}}}}} \right)}\end{equation*}

Converting MPB using this approach served as a consistent visual and quantitative proxy for density while reducing the potential for error in the outermost edges of the grayscale range. These outermost areas in question are more variable, uncertain, and contain questionable data with artifacts of scanning (e.g., cheesecloth, skin, and other matter). The MPB of the 25% and 75% phantoms for each scanned species and the resulting 10% and 90% grayscale colormap values can be found in Supplemental Table 1. In addition to analysis of the CaHA values, images were captured of each micro-CT scan set to these calculated colormap ranges. Images were then qualitatively compared for bone density variation across macrourids.

### Phylogenetic reconstruction, phylogenetic signal, and ancestral character-state reconstruction

To address the influence of species relatedness on observed bone density variation, a phylogeny of rattail fishes was inferred from one nuclear (RAG1) and two mitochondrial (COI, 16S) gene sequences. Previously published sequences were pulled from GenBank that were published in the following studies: Yamaguchi et al. (2000); Smith and Wheeler (2006); Campo et al. (2007); Miya et al. (2007); Roa-Varón and Ortí (2009); Fukui et al. (2010); Zhang and Hanner (2011); Near et al. (2012); Wainwright et al. (2012); Grande et al. (2013); Laptikhovsky et al. (2013); Gaither et al. (2016); Oliveira et al. (2016); Chang et al. (2017); Robertson et al. (2017); Martin et al. (2018) and additional unpublished barcoding studies indicated in GenBank and found in Table 1. Taxonomic sampling for the phylogenetic dataset included 12 of the 15 rattail species used in this study and three additional teleosts (Myctophidae, Polymixiidae, and Merlucciidae) as the outgroup (Table 1). The data matrix analyzed was 85.6% complete and contained 411 informative sites. Sequences were aligned with MAFFT (Katoh and Standley 2013) and were concatenated and trimmed by eye in Mesquite (Maddison and Maddison 2019). Tree estimation and model testing were performed in IQ-TREE (Nguyen et al. 2015) rooted with *Lampichthys preocerus*, and data were partitioned by gene. Substitution model testing for each gene was performed through IQ-TREE using modelfinder and the BIC criterion (Kalyaanamoorthy et al. 2017). Substitution models were assigned as follows: COI was assigned HKY + F + I + G4, 16S was assigned TIM2e + G4, and RAG1 was assigned HKY + F + G4 (Chernomor et al. 2016). Likelihood analysis for the phylogenetic tree was performed in IQ-TREE and bootstrap analysis used ultrafast bootstrapping (Hoang et al. 2018) for 1000 replicates.

To assess phylogenetic signal of bone density across rattails, the “phylosig” function from *phytools* (Revell 2012) and *ape* (Paradis and Schliep 2019) in the software program R (R Core Team 2018) were used. *P*-values were based on 1000-iteration permutations. An additional maximum-likelihood ancestral character-state reconstruction was performed in R using the “anc.ML” function from *phytools*. Two outgroup genera, *Polymixia* and *Lampichthys*, were trimmed from the tree prior to analysis.

### Statistical analyses

A two-way analysis of variance (ANOVA) was performed in R using the function “aov” from the base *stats* package on the average bone densities among species of macrourid, among bones within a species (lower jaw, eleventh vertebrae, pelvic girdle, first pterygiophore), and by number of years since collection. Length of time in unbuffered formalin is thought to increase the amount of demineralization that occurs prior to storage in alcohol. These museum collections lack information regarding the length of time each specimen spent in formalin, but Fonesca et al. (2008) found that after fishes were immersed in formalin for one day, the only significant difference between bone densities was between day one and any time after 30 days, suggesting that specimens kept in formalin for any period of time upwards of a month are unlikely to show significant density changes among specimens due to fixation processes. Additionally, Fonseca et al. (2008) found no significant difference between bone mineralization in fishes that were fixed in 10% unbuffered, 10% formalin phosphate buffered, and 10% calcium-carbonate buffered formalin. Specimen date of collection can be found in Table 1.

To control for covariation due to species relatedness, phylogenetic generalized least squares (PGLS) models were used to analyze the effect of maximum observed habitat depth, depth at capture, and specimen pre-anal fin length (PAF) on bone density using the “gls” function in R. This function assesses whether a particular trait is influenced by how closely related individuals are in a given phylogeny. If the assessed trait shows a phylogenetic signal, PGLS will correct trait values based on the strength of that signal. Habitat depths were based on values given for species occurrences from Cohen et al. (1990), Iwamoto and Sazonov (1994), Iwamoto and Schneider (1995), Hoff et al. (2015), Linley et al. (2016), and Cruz-Acevedo and Aguirre-Villaseñor (2020). Pre-anal fin length is commonly used instead of total length or standard length in rattail studies because specimen tails can be easily damaged in life or during collection (Andrews et al. 1999). Total length measurements of fishes with damaged tails would skew results. Additionally, rattails can regenerate their tails, and regenerated tails may not reach their initial total length, potentially confounding length-based analyses. Thus, age and growth curves are better represented using pre-anal fin length in rattails (e.g., Andrews et al. 1999; Gerringer et al. 2018). When a species included in the analyses is represented by multiple specimens in the study (e.g., *Coryphaenoides anguliceps* Garman 1899*, C. yaquinae*), data from the larger specimen was chosen for phylogenetic-associated analyses. We also performed additional general linearized models associated with density and pre-anal fin length. Information on pre-anal fin length, depth of capture, and maximum observed habitat depth can be found in Table 1.

## Results

**Fig. 3:**
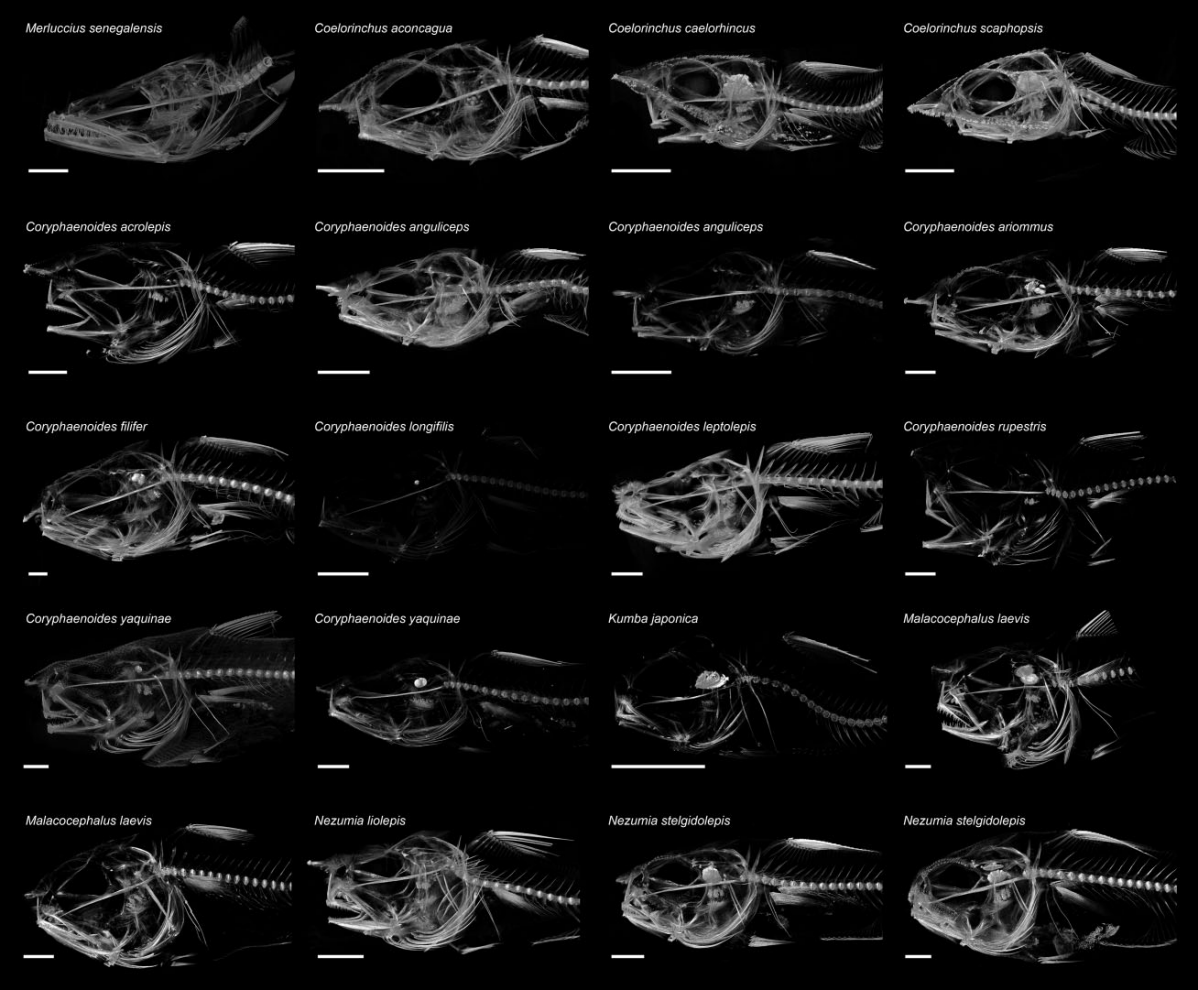
Micro-CT scans of the macrourid specimens used to examine bone density in this study, ordered alphabetically by genus and species. Images standardized to show 10% – 90% calcium hydroxyapatite for use in qualitatively comparing variation in bone density. Scale bars represent 10 mm.

In this study, we micro-CT scanned 20 specimens representing 15 species of rattail to assess patterns of bone density variation related to buoyancy, finding differences among bones within a specimen and among bones across species. Rattail micro-CT scan images in Fig. 3 were configured with Amira colormap settings that visually reflect differences in bone density, with brighter values portraying higher densities. Prominent differences are seen in the lower brightness values of *Coryphaenoides longifilis* Günther 1877 and *Kumba japonica* (Matsubara 1943) and the higher brightness values of *Coryphaenoides leptolepis* Günther 1877 and *Coelorinchus scaphopsis* (Gilbert 1890; Fig. 3)*.*

**Fig. 4:**
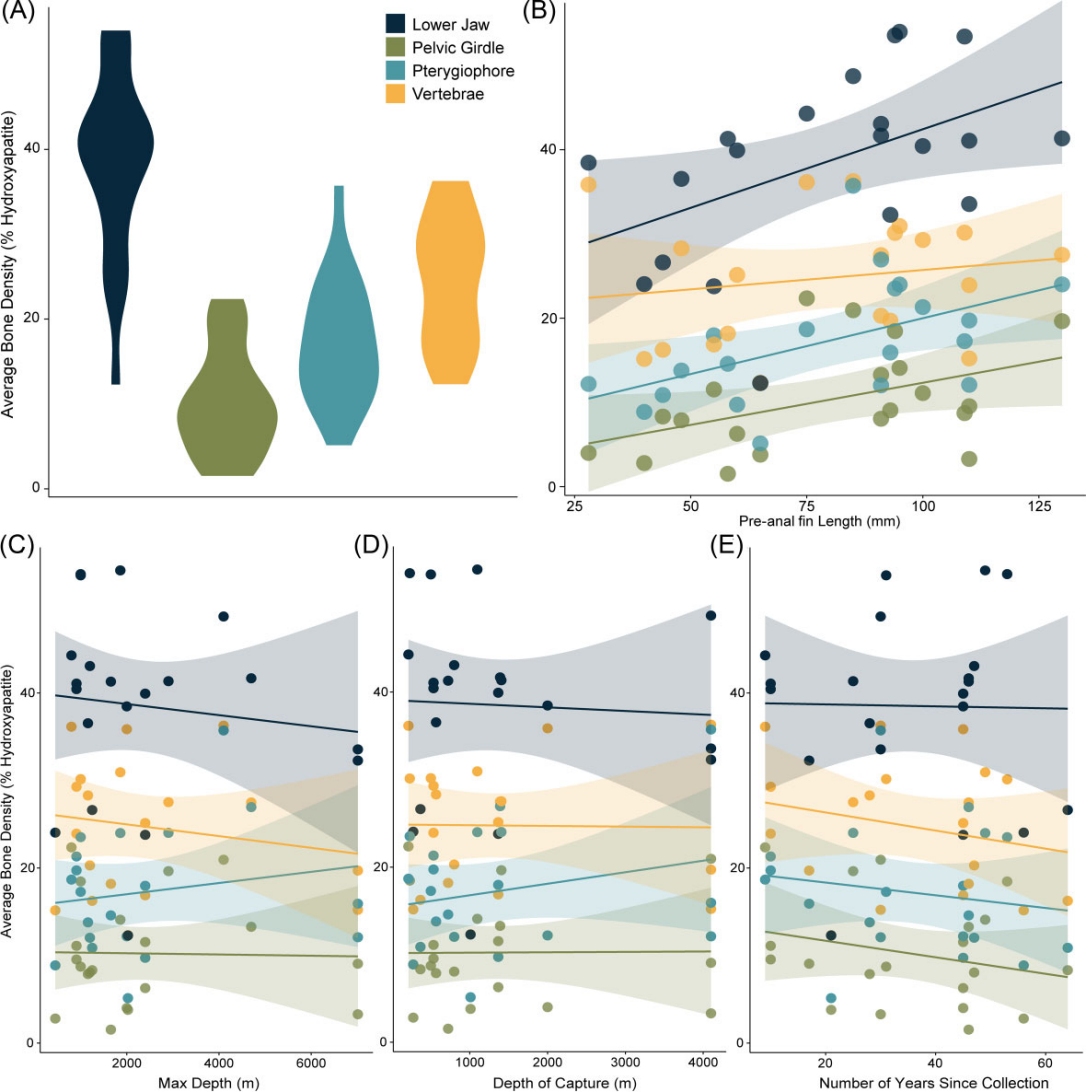
**(A)** Bone density variation of the lower jaw, pelvic girdle, first pterygiophore, and 11th vertebrae across 20 specimens of rattails. Comparison of average bone density with a specimen's **(B)** pre-anal fin length, **(C)** maximum depth, **(D)** depth at capture, and **(E)** years since collection.

Bone density varied considerably among different bones within a specimen and among species (Fig. 4; Tables 2 and 3). Overall, the lower jaw was significantly denser than all other assessed bones (*p* < 0.001), and the pelvic girdle was significantly less dense than all other assessed bones (*P* < 0.001; Fig. 4A Table 3). When comparing across species, the densest lower jaws were found in *Coryphaenoides ariommus* Gilbert and Thompson 1916 (54.0% hydroxyapatite) and *Malacocephalus laevis* (Lowe 1843; 32353.6% and 53.4% hydroxyapatite), while *Coryphaenoides longifilis* (12.3% hydroxyapatite) and *Coryphaenoides anguliceps* (23.8% hydroxyapatite) had the least dense lower jaws; Figs. 3 and 4; Table 2). Density values for all bones and all specimens can be found in Table 2. *Merluccius senegalensis* (lower jaw density: 44.3% hydroxyapatite), the near-shore outgroup species in the closely related family Merluccidae, presented a similar trend in bone density variation to those of the Macrouridae, with a dense lower jaw (44.3% hydroxyapatite) compared to the other assessed bones, and a pelvic girdle with lower density (Table 2).

**Table 2:** Specimens used in this study along with their museum ID, pre-anal fin length, and average hydroxyapatite per assessed bone. Hydroxyapatite percentages calculated from voxel brightness of rattail CT scans with known phantom densities.

Genus	Species	Museum ID	Pre-anal fin length (mm)	Lower jaw (%CaHA)	Pelvic girdle (%CaHA)	1st pterygiophore (%CaHA)	11th vertebra (%CaHA)
*Coelorinchus*	*aconcagua*	SIO 65-675	40	24.05	2.8	8.88	15.16
*Coelorinchus*	*caelorhincus*	SIO 65-367-62A	44	26.63	8.32	10.89	16.25
*Coelorinchus*	*scaphopsis*	SIO 68-90	48	36.54	7.89	13.79	28.29
*Coryphaenoides*	*acrolepis*	SIO 71-141	91	41.67	13.29	26.96	27.5
*Coryphaenoides*	*anguliceps*	SIO 59-265	55	23.79	11.54	17.97	16.88
*Coryphaenoides*	*anguliceps*	SIO 59-265	60	39.92	6.28	9.74	25.14
*Coryphaenoides*	*ariommus*	SIO 72-183	95	54	14.09	24	30.94
*Coryphaenoides*	*filifer*	UW 41684	130	41.35	19.64	24.02	27.52
*Coryphaenoides*	*leptolepis*	SIO 91-152	85	48.74	20.95	35.72	36.27
*Coryphaenoides*	*longifilis*	UW 119661	65	12.3	3.8	5.13	12.33
*Coryphaenoides*	*rupestris*	SIO 74-183	91	43.1	8.06	12.03	20.3
*Coryphaenoides*	*yaquinae*	SIO 04-101	93	32.28	9.07	15.92	19.71
*Coryphaenoides*	*yaquinae*	SIO 91-145	110	33.55	3.29	12.09	15.23
*Malacocephalus*	*laevis*	SIO 79-344	94	53.58	18.46	23.53	30.13
*Malacocephalus*	*laevis*	SIO 00-200	109	53.44	8.73	17.28	30.16
*Merluccius*	*senegalensis*	CAS 235494	75	44.29	22.36	18.68	36.15
*Nezumia*	*japonica*	SIO 77-157	28	38.46	4	12.2	35.86
*Nezumia*	*liolepis*	SIO 71-1	58	41.3	1.54	14.59	18.19
*Nezumia*	*stelgidolepis*	UW 153473	100	40.44	11.11	21.32	29.29
*Nezumia*	*stelgidolepis*	UW 153474	110	41.07	9.56	19.73	23.93

**Table 3:** Results from the PGLS and ANOVA. Stars denote significant values. Collection depth, maximum depth, and pre-anal fin length were tested independently. Akaike information criterion (AIC) values are reported for each model. The metric with the lowest AIC value for each bone appears in bold type. The p-values below an alpha threshold of 0.05 are marked with an asterisks and bold type.

	Collection Depth			Max Depth			Pre-Anal Fin Length		
PGLS Density	Coefficient	*P*-value	AIC	Coefficient	*P*-value	AIC	Coefficient	*P*-value	AIC
**Lower Jaw**	0.00286	0.5839	134.1	-0.00012	0.9714	134.5	0.6436	*0.0009	**120.9**
**Pelvic Girdle**	0.001773	0.1852	97.76	0.000596	0.5052	99.38	0.1394	*0.0145	**92.55**
**Pterygiophore**	0.003668	*0.0393	103.6	0.001425	0.2482	107.2	0.1929	*0.0169	**101.8**
**Vertebrae**	0.001943	0.3382	109.1	0.000346	0.7965	110.2	0.2342	*0.0036	**99.77**
** **	*P*-values			Coefficients					
ANOVA Density	Lower Jaw	Pelvic Girdle	Pterygiophore	Lower Jaw	Pelvic Girdle	Pterygiophore	Vertebrae		
**Pelvic Girdle**	< 0.001			38.52	10.24	17.22	24.76		
**Pterygiophore**	< 0.001	0.009							
**Vertebrae**	< 0.001	< 0.001	0.01						
	Pre-Anal Fin Length								
GLM Density	Coefficient	*P*-value	AIC						
**Lower Jaw**	0.18659	*0.0312	151.5			** **	** **		
**Pelvic Girdle**	0.09998	*0.048	130.5			** **	** **		
**Pterygiophore**	0.13249	*0.0223	135.1			** **	** **		
**Vertebrae**	0.04596	0.4761	124.2			** **	** **		

### Bone density by depth, length, and collection date

Results from the PGLS suggest no relationship between bone density and collection depth or maximum depth of the species (Fig. 4C, D; Table 3). However, we found a significant correlation between bone density and pre-anal fin length for all assessed bones (*P* < 0.01; Fig. 4A; Table 3). Lower jaw density increased by approximately 2% for every 10 mm increase in pre-anal fin length, approximately 1% for the pelvic girdle, approximately 1% for the pterygiophore, and approximately 0.5% for the vertebra (Fig. 4B). Additionally, we found no significant relationship between bone density and length of time since preservation (Fig. 4E; Table 3).

Although there is significant variability in bone density across the rattail species in this study, we found no relationship between bone density and maximum observed habitat depth or habitat depth at capture (all *P* values > 0.05; Table 3; Fig. 4). Our specimens of the deepest living species *Coryphaenoides yaquinae* (collection depth 4,100 m), had lower jaw densities of 32.3% and 33.6% hydroxyapatite, respectively, and *C. leptolepis* (collection depth 4,100 m) with a lower jaw density of 48.7% hydroxyapatite (Table 2; Fig. 4). However, we found that bone density significantly increased with increasing length across all but one rattail species (Fig. 4D, *P* < 0.01). *Coryphaenoides yaquinae* (Fig. 4D) was the exception to this trend and had exceptionally low bone densities.

### Macrourid phylogeny, phylogenetic signal, and ancestral character-state reconstruction

**Fig. 5:**
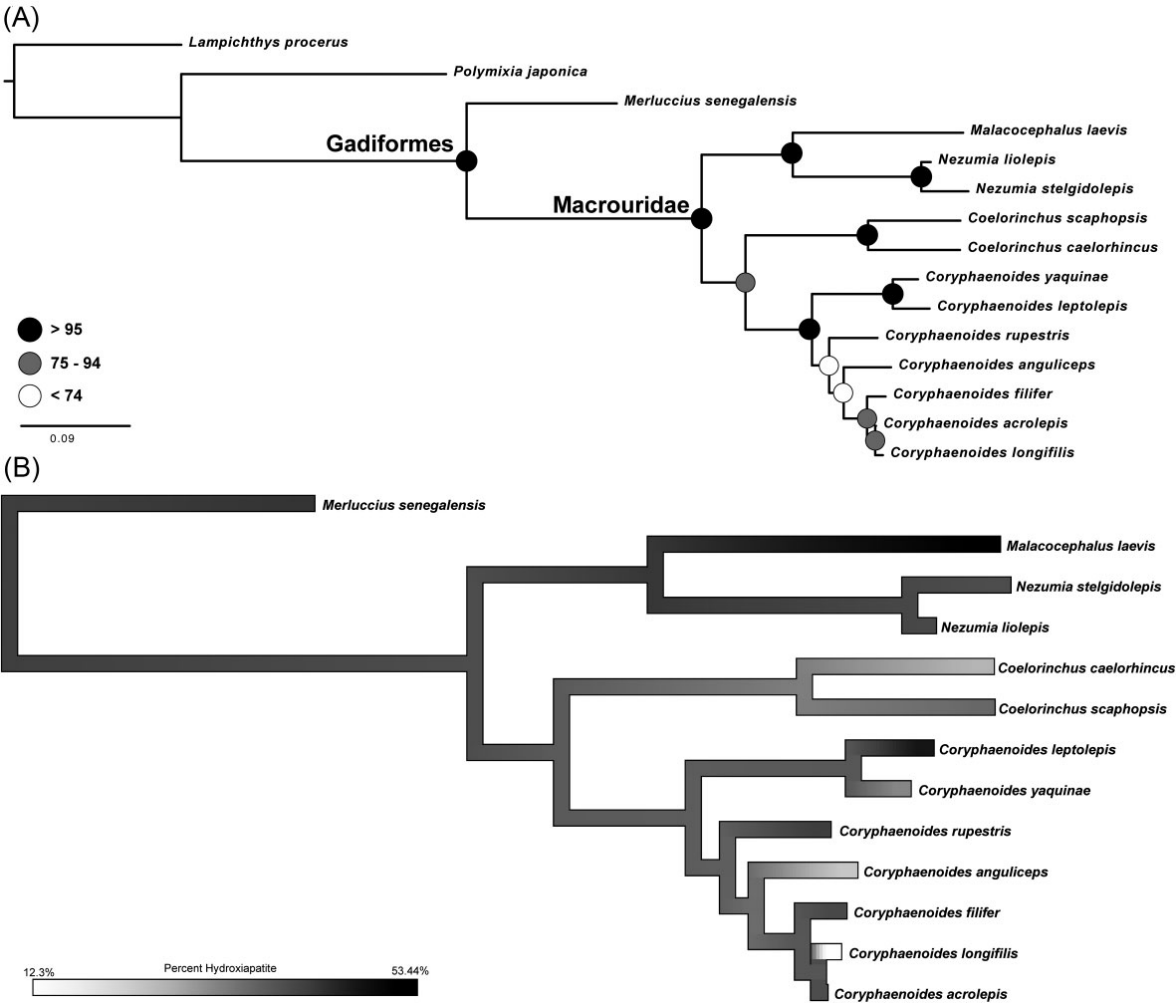
**(A)** Maximum-likelihood phylogeny of rattail taxa used in this study with available sanger sequence data on Genbank. Scale bar represents the number of substitutions per site and bootstrap support values shown at nodes. **(B)** Ancestral character-state reconstruction of lower jaw bone density mapped along the branches of the phylogeny. Color map corresponds to percent hydroxyapatite.

We conducted a phylogenetic analysis to address the influence of phylogeny on variation in bone density. We were able to include 12 of the 15 macrourid species used in this study. The family Macrouridae was recovered as monophyletic (Fig. 5) with high support (bootstrap values >95). All genera were recovered as monophyletic with high support. *Nezumia* was recovered as monophyletic and sister to *Malacocephalus* with high support. The *Nezumia* + *Malacocephalus* clade was recovered sister to a clade containing *Coelorinchus* + *Coryphaenoides* (Fig. 5). Rattail relationships from our phylogenetic analysis corroborate those of Gaither et al. (2016). There was low phylogenetic signal in average bone density for all rattails in this study, but all *P*-values were non-significant, suggesting the data provided do not give enough information to indicate anything other than random processes of evolution. The ancestral character-state reconstruction of bone density variation across the rattail phylogeny also suggests no correlation between bone density and phylogeny (Fig. 5). The species with the highest bone density *Coryphaenoides acrolepis* (Bean 1884) was resolved as sister to the species with the lowest bone density (*C. longifilis*), and bone density values across the rattail tree shows no pattern.

## Discussion

Our micro-CT data show that all the macrourids examined in this study possess similar patterns of variability in bone density across all four bones. Contrary to the prevailing hypothesis, we found no relationship between bone density and maximum habitat depth or depth at capture for these 15 species (Priede, 2018). Instead, we find a significant positive relationship between rattail length and bone density, suggesting that as a fish gets bigger their bones get denser (Fig. 4; Table 2). Variation in skeletal mineralization of deep-sea fishes has previously been tied to buoyancy adaptations (Eastman and Devries 1982; Gerringer et al. 2021b). Our assessment of bone mineralization in rattails suggests a different association in this group. The present study has some limitations. The number of specimens and species was limited due to the rarity of some deep-sea rattail species, limiting the statistical strength associated with our findings. Even in this select sample of species, we find significant trends in bone-density variation. Additionally, selecting a targeted small number of bones for analysis has limiting factors. The specific bones chosen for analysis were analyzed across all specimens, but we cannot exclude the fact that some of the bone-density variation we observed may be due to slight differences in life history across species. With these limitations in mind, we introduce the following discussions associated with our observed variation in bone mineralization in rattails.

### Buoyancy in rattails

A reduction in the usefulness of gas-filled swim bladders for maintaining buoyancy under the presence of high hydrostatic pressure in deep-sea environments has given rise to the hypothesis that fishes may be decreasing high-density tissues (i.e., bone) to increase buoyancy in the deep sea. We found no relationship between bone mineralization and depth across our sample of rattails. Additionally, almost every species of rattail still possesses a swim bladder. Approximately 13 species, including *Albatrossia pectoralis* (Gilbert 1892)*, Echinmacrurus*, and two species in the Macrouroidinae, possess either regressed swimbladders or lack them completely (Cohen et al. 1990). Loss of swim bladders in these species may be tied to alternative methods of buoyancy or differences in life history. *Albatrossia pectoralis* has extremely high-water content in its muscles (Crapo et al. 1999), and the two species in the Macrourodinae are hypothesized to be bathypelagic (Marshall 1965; Marshall and Tåning 1966) and have reduced organ systems (Marshall 1960). We found no correlation between bone density and maximum depth or depth at capture across the 15 rattail species assess in our study. Previous studies show that many deep-sea fish lineages that retain their swim bladder generally lack adaptations related to buoyancy seen in lineages that have lost their swim bladders. Bristlemouths (gonostomatids) have a reduced skeleton and muscle tissue or have oil-filled swim bladders (Blaxter et al. 1971; Crabtree 1995; Priede 2017). Squaretails (tetragonurids) have reabsorbed their swim bladder (Priede 2017), and some barreleye species *Dolichopteroides binocularis* (Beebe 1932) have evolved enlarged pelvic fins for floating in the water column. Although there are a myriad of adaptations for buoyancy in deep-sea fishes lacking swimbladders, it is less costly than might be expected to retain and use a gas-filled swim bladder under the extreme hydrostatic pressures of the deep sea. For marine fishes to be weightless in water, the swim bladder should have a volume equal to ∼ 5% of the animal's total volume (Jones and Marshall 1953). Energetically, it becomes increasingly costly to inflate the swim bladder in deeper waters due to high hydrostatic pressure, but less so than producing and sequestering lipids to maintain the same amount of buoyancy. It seems unlikely for natural selection associated with buoyancy to result in the loss of the swim bladder in a species of deep-sea fish while, at the same time, evolving something else entirely (Priede 2018). McCune and Carlson (2004) found that the loss of the swim bladder is common in wild-caught zebrafish and occurs due to multiple genetically distinct mutations. They also found that, at a minimum, the swim bladder has been lost approximately 32 times within teleosts and up to at least 57 times if multiple independent losses within a family are included. Loss of the swim bladder appears to be frequent at the genetic level, so maintenance of swim bladders in rattails is likely being selected for. The males of many rattail species also have accessory muscles attached to their swim bladders used in “drumming” (Marshall 1965), muscles not found in the deep-living *Coryphaenoides*. Having multiple uses for the swim bladder would increase the likelihood that this organ is being maintained in rattails.

Even though swim bladders remain useful under high hydrostatic pressures, their functionality may still be reduced in the deep sea. Rattails may be enhancing the use of their swim bladders by incorporating additional adaptations. Of the four bones assessed in this study, we find the lowest bone density in the pelvic girdle, and the pelvic fins of rattails are not known to be actively used in swimming (Greek-Walker and Pull 1975). Previous work found chemosensory cells on fin-rays of other gadiforms (Whitear and Kotrschal 1988), suggesting that similar chemosensation could be occurring throughout the order and is worth assessing on macrourid pelvic fins. Pelvic fins are thought to be one of the most frequently lost features of teleosts and are hypothesized to have been lost at least 50 independent times and up to 92 times or more (Nelson 1989–1990). If, at the species level, rattails are differentially reducing mineralization in their skeleton, it would likely occur in lesser used structures such as the pelvic girdle. Within rattails, there is a complete loss of pelvic fins in *Macrouroides*, and greatly reduced pelvic fins in *Squalogadus* (Marshall and Tåning 1966; Priede 2017). Ross and Gordon (1978) analyzed multiple species of rattails, including *Coryphaenoides rupestris* Gunnerus 1765 and *Coelorinchus caelorhincus* (Risso 1810), and uncovered a trend of increasing guanine crystals in deeper-living species. Under high pressures, large quantities of guanine crystals may decrease the permeability of gasses through the swim-bladder membrane, helping retain gas and reducing the energetic cost of maintaining an inflated swim bladder. Retention of the swim bladder in deep-sea fishes is not uncommon and is seen in deep-sea cusk-eels (ophidiids, caught at 7160 m; Nielsen and Munk 1964). One of the deepest-living fish species that still retains the use of their swim bladders is the rattail species *Coryphaenoides yaquinae* (Linley et al. 2016). Additionally, many rattail species have restricted depth ranges. These fishes are not performing long migrations across the sea floor to shallower depths or regularly moving large vertical distances in the water column. This restriction of depth reduces the need to routinely change the volume of gas sequestered in the swim bladder (Cohen et al. 1990). Although some of the deeper-dwelling species have wider depth ranges than their shallower-water relatives (Fig. 1), recent research by Gaither et al. (2018) shows that within Atlantic populations of *Coryphaenoides rupestris* there exists deeper and shallower water specialists with distinct genotypes at 1000 m and 1800 + m. Juveniles of these populations sort by habitat as they mature. If these findings are reflected in even a small portion of other deep-sea rattail species, they may be behaving similarly to shallower species that exhibit more restricted ranges and are stratifying by depth.

We found no relationship between bone mineralization and depth across our sample of rattails, but these fishes may be reducing density in other tissues. Crabtree (1995) and Tuponogov et al. (2008) found that, similar to some mesopelagic fishes like alepocephalids (Priede 2017), some rattails exhibit reduced density via the development of soft watery muscles. Rattails are one of the few benthopelagic lineages studied that, while retaining their swim bladders, also showed an increase in the water content of their muscles. Crabtree (1995) suggested this could result in more efficient growth at depth tied to a decrease in energy input needed to produce a given body size. We suggest this could also be related to increasing buoyancy. Additionally, Gerringer et al. (2017a) found gelatinous tissue in *Coryphaenoides yaquinae* that was buoyant in seawater, the main function of which was thought to be aiding in buoyancy. Gerringer et al. (2017a) assessed only a handful of rattail species in their study and found this tissue-type in one species. There are 369 described species of rattails, so this type of buoyant gelatinous tissue may be more widespread across the group. The combination of these findings, in addition to our own, suggest that deep-sea rattails are using a variety of adaptations to remain buoyant under high hydrostatic pressure. Although we do not see a clear trend of selection against non-adaptive skeletal structures or reduction of the density of certain bones associated with increasing depth in rattails, we do see what could be a complex system where very specific adaptations associated with buoyancy occur mainly at the species level. If different species are evolving a variety of adaptations to living in increased hydrostatic pressures, then the variability we find in skeletal density across the group may suggest a more apomorphic trend and that demineralization of the skeleton in specific species has evolved as a supplement to other buoyancy adaptations.

### Demersal and benthopelagic comparisons

Specific environmental conditions and natural selection can drive the evolution of convergent adaptations among disparate lineages of animals. Many of the fish lineages that have lost swim bladders and may need other adaptations to maintain neutral buoyancy are also associated with benthic or deep-sea lifestyles (McCune and Carlson 2004). A recent study by Gerringer et al. (2021) analyzed skeletal density in the snailfishes (family Liparidae), a mainly demersal/benthic lineage, using micro-CT scanning methods. Most snailfishes lack swim bladders and were found to have reductions in structural dimensions and loss of skeletal elements with increasing depths. Gerringer et al. (2021b) additionally found skeletal differences among the few pelagic species compared to the demersal species, where pelagic species had lower numbers of vertebrae and lower bone density than their demersal relatives. Alternatively, most rattails are benthopelagic and regularly inhabit the water column (Iwamoto and Schneider 1995). Lifestyle and habitat differences (i.e., pelagic vs. demersal/benthic) are thought to be a major driver behind the variation in adaptations tied to buoyancy in fishes. Lineages that have retained their swim bladders and continue to use the water column may rely more heavily on adaptations associated with the swim bladder itself. Other lineages like the snailfishes that have more benthic life histories, are more closely associated with the seafloor, and have lost their swim bladders, may rely more heavily on other adaptations to maintain buoyancy, including reduction or loss of skeletal structures. Additional insights may be gained by looking at other lineages occupying similar bathymetric ranges and which have similar lifestyles (e.g., cusk eels (Ophidiidae) and eelpouts (Zoarcidae)). The intricacies behind skeletal mineralization and other anatomical changes across all fishes are arguably immensely complicated and could be tied to a wide variety of factors other than as an adaptation to buoyancy, including ontogeny, feeding, and locomotion.

### Body size and feeding

While we did not see a decrease in density with an increase in depth, we did find increased mineralization of the skeleton in larger rattail species and in larger individuals within a species. There are numerous studies across a wide range of taxa focused on osteological development and adaptations of fishes growing from larvae to adults (e.g., Matsuoka 1985; Brown et al. 2001; Murray 2004; Rutledge et al. 2019). It is hard to study these developmental aspects in most rattail species due to limited survey data for various life stages in many of these species (Endo et al. 1992; Busby 2005). Most rattails are believed to have pelagic eggs and larvae which migrate to deeper waters through larval, juvenile, and adult stages (e.g., Fukui et al. 2010; Lin et al. 2012). Gas glands can be found in the larval forms of these species (Matarese et al. 1989), many of which are inhabiting different pressure regimes throughout their lives and where retention of the swim bladder may be the most viable way to maintain buoyancy. Our findings suggest that bone density may increase in rattails through ontogeny, not only during the larval and juvenile stages, but also during adulthood. When accounting for phylogeny, this trend is significant for each bone analyzed in this study (Table 3). Increasing mineralization of the skeleton throughout adulthood could be tied to multiple factors. Vertebrae are important support structures for muscle attachments and swimming in fishes. Hamilton et al. (1981) analyzed the mechanical properties of vertebrae in three species of freshwater fishes at different ages. In all three species, they found an increasing trend of bone density with age and length. Increases in bone density, though, were not necessarily associated with “strength,” and variation in the ratios of organic and inorganic matrices determined how strong vertebrae were at each age (Hamilton et al. 1981). Rattails are very efficient, slow, undulatory, swimmers and use the epaxial and hypaxial muscles (myotomes) that are anchored to the vertebral column during swimming (Neat and Campbell 2013). Myotomes become larger as individuals increase in size and may need increased structural support via increased density of the bone matrix. Similarly, pterygiophores provide attachment points and support structures for fish dorsal fins. We found increasing density with increasing specimen length in the first pterygiophore of the 15 rattail species in this study. Rattails have small, but tall, first dorsal fins. Increasing the structural integrity of the first pterygiophore would provide necessary support for the increasing size of the dorsal fin with increasing size of the specimen. Lastly, an increase in the bone density of the lower jaw throughout adulthood could be tied to feeding.

Rattails are known to feed on a wide range of organisms and, depending on the species, have been categorized as micronektonivores, crustacean feeders, and piscivores (Drazen and Sutton 2017). Similar to many deep-sea demersal fishes, they are also facultative scavengers, eating whatever they can find that is available. Drazen et al. (2008) found that abyssal macrourids *Coryphaenoides armatus* (Hector 1875) and *C. yaquinae* are top predators on benthic fauna but also derive much of their nutrition from larger epipelagic organisms that sink as carrion. Given the opportunity, these rattails scavenge, and carrion may be a more vital resource than benthic prey. Dense jaws enable feeding on carcasses of larger organisms from the epipelagic and would play a large role in a rattail's ability to be both a predator and scavenger. Studies looking at zebrafishes show increased mineralization due to changes in water flow conditions, similar to how physical activity promotes gain in bone mass and lack of mechanical loading results in resorption in vertebrates (Suniaga et al. 2018). Analogous to the findings in this study, Denton and Marshall (1958) noted that in *Gonostoma*, a pelagic fish genus with species that exhibit reduced bone mineralization, have watery muscles, and impaired swimming performance, maintained highly mineralized jaws that retained full function. Although we do not see a trend of demineralization of the skeleton with depth, we do find that the lower jaw was significantly denser than the other bones assessed in this study and had the largest increase in density associated with specimen length. The necessary use of the oral jaw bones for feeding suggests there may be a constraint on reducing bone density in the jaw compared to more underutilized bones in rattails.

## Conclusions

Our findings suggest that although rattails exhibit variation in the density of their bones (Figs. 3 and 4; Table 2), that variation is not altogether correlated with buoyancy and under high hydrostatic pressures with increasing depth (Fig. 4). Rattails are likely using a variety of adaptations to increase buoyancy at great habitat depths, including evolving lower density muscles, guanine crystals in their swim bladders, and buoyant gelatinous tissues. Bone demineralization may be occurring on a more case-by-case species level to enhance the usefulness of the swim bladder. Additionally, the low-density pelvic girdles of our 15 assessed species could point to reducing mineralization in comparatively unused bones. We do find a significant correlation between pre-anal fin length and average bone density (Fig. 4B), where density increases with increasing specimen length. Increased density of the vertebrae and first pterygiophore may be tied to structural support and muscle attachment points while increasing density in the lower jaw is likely tied to feeding, especially since rattails are known to be opportunistic scavengers on carcasses. Ultimately, the functional implications behind variation in bone density across the rattails is complex and likely tied to range of behavioral, environmental, and life history factors.

Future work in this area should focus on increasing the taxonomic sampling of rattail species. Although this study includes specimens from a wide range of habitat depths and from the largest genus of rattails (*Coelorinchus*, ∼122 spp*.*), it lacks species from a large portion of the macrourid phylogeny (Roa-Varón and Orti 2009). Additional focus on assessing the differences in bone densities among macrourid species that are known to be more pelagic (e.g., *Cynomacrurus, Nezumia parini* Hubbs & Iwamoto 1977), or have extremely reduced or missing swim bladders (*Echinmacrurus*, *Macrouroides, Squalogadus*, *Albatrossia*) than the more common benthopelagic species that have retained their swim bladders. The few species that have reduced or lost their swim bladders may show a trend in bone demineralization, similar to other lineages of fishes that have adapted similar strategies to staying buoyant in the water column.

## Data Availability

The data collected for this study are available in the supplementary data section.

## Supplementary Material

obac044_Supplemental_Table
